# ADULT CHILDREN'S EDUCATIONAL ATTAINMENT AND PARENT HEALTH IN MID- AND LATER LIFE

**DOI:** 10.1093/geroni/igac059.1170

**Published:** 2022-12-20

**Authors:** Christopher Dennison, Kristen Schultz Lee

**Affiliations:** University at Buffalo, SUNY, Buffalo, New York, United States; University at Buffalo, SUNY, Buffalo, New York, United States

## Abstract

While intergenerational models of adult health contend that children’s educational attainments influence the health of their parents, background characteristics that predict both can confound the results. Data from the National Longitudinal Study of Adolescent to Adult Health Parent Study are used to examine how having no children who completed college influences parents’ self-rated health and depressive symptoms. We use propensity scores to assess this relationship net of potential confounders and test for heterogeneity in the consequences associated with having no children who completed college. Having no children who completed college is negatively associated with parents’ self-rated health and positively associated with depressive symptoms. Among parents with the highest propensity for having no children who complete college, the consequences on depressive symptoms are greatest. These findings are important given the call for investments in children’s educational opportunities as a vehicle for promoting health among adults and their older parents.

